# Kinetic Investigation of Photocatalytic Degradation of Methyl Orange Dye Using Mg‐Doped Ag_2_O Nanoparticle

**DOI:** 10.1155/sci5/8874791

**Published:** 2026-02-12

**Authors:** Gul Asimullah Khan Nabi, Sajjad Hussain, Mati Ullah, Shohreh Azizi, Ilunga Kamika, Malik Maaza

**Affiliations:** ^1^ Department of Chemistry, Bacha Khan University, Charsadda, 24420, Khyber Pakhtunkhwa, Pakistan, bkuc.edu.pk; ^2^ Department of Physics, Abdul Wali Khan University Mardan, Mardan, 23200, Khyber Pakhtunkhwa, Pakistan, awkum.edu.pk; ^3^ UNESCO-UNISA Africa Chair in Nanosciences/Nanotechnology, College of Graduate Studies, University of South Africa, P.O. Box 392 Muckleneuk Ridge, Pretoria, South Africa, unisa.ac.za; ^4^ Institute for Nanotechnology and Water Sustainability, College of Science, Engineering and Technology, University of South Africa, Florida, Johannesburg, 1709, South Africa, unisa.ac.za

**Keywords:** double-distilled water, kinetic and thermodynamic studies, methyl orange, Mg-doped Ag_2_O NPs

## Abstract

In the current research, the Mg‐doped Ag_2_O nanoparticles were produced by the coprecipitation method. The characterization of the as‐prepared nanoparticles was conducted through a series of techniques. SEM confirmed homogenous morphology of a sphere with minimal agglomeration. The band gap of Mg–AgO_2_ was determined to be 2.6 eV. The materials were observed to possess a particle dimension of 23.87 nm. X‐ray diffraction (EDX) proved that the substance is 97% Ag, 1.58% oxygen, and 1.42% Mg. The FTIR, PL, DRS, PZC, TEM, and XRD results proved that Mg‐doped Ag_2_O nanoparticles were successfully doped. In this study, the first application of the synthesized Mg‐doped Ag_2_O particles was studied for the decolorization of methyl orange in an aqueous solution. At ideal conditions of dye (30 ppm), neutral pH and the dosage of catalyst (0.4 g), 96.1% of decolorization was obtained in 150 min. The degradation was first order and the activation energy was found to be 13.19 kJ/mol. The recyclability of the material was studied, and it was confirmed that the material could be reused multiple times.

## 1. Introduction

Industrial wastewater pollution is considered the major world environmental challenge, especially in areas that are dealing with rapid industrialization and​ water scarcity [1, 2].

Industries include food manufacturing, paper, and textiles, which generate a high volume of dye‐containing wastewater [3–6]. Dyes are highly hazardous chemicals, which are nonbiodegradable [7]. Among various pollutants, methyl orange (MO) is generally used in the textile industry and poses significant environmental threats [8]. Thus, it is required that it be removed from wastewater.

Although several treatment techniques have been studied, photocatalysis has been shown to be the ideal approach for the removal of organic dyes [9, 10]. In this process, the organic pollutants are totally decomposed at low cost and without any toxic substances being released into the environment [11, 12]. TiO_2_ and ZnO are considered as conventional photocatalysis that have been extensively studied due to their availability and stability [13–15]. However, their wide band gaps limit their capability and effectiveness under visible light [16]. On the other hand, silver oxide (Ag_2_O), with a lower band gap and strong visible‐light absorption capacity, has become an excellent candidate for photocatalytic activity [17, 18]. However, the stability and activity of Ag_2_O are reported as major drawbacks in photocatalytic applications [19, 20]. Various modification techniques have been used to increase the performance of metal oxides to address these drawbacks [21, 22]. In order to address these tackles, the most effective approach has been proposed to be metal doping, which can alter the band gap and improve charge separation [19, 23, 24]. This study aims to synthesize Mg‐doped Ag_2_O nanoparticles via the coprecipitation method and their application in the photocatalytic degradation of MO.

## 2. Experimental Section

### 2.1. Materials

Reagent‐grade silver nitrate (AgNO_3_, ≥ 99.9%, Cat. No. 3456456) and magnesium nitrate hexahydrate (Mg(NO_3_)_2_·6H_2_O, ≥ 99%, Cat. No. M8266) were obtained from Sigma‐Aldrich. Reagent‐grade MO (MW = 327.33 g/mol, ≥ 99%, Cat. No. 32456) and sodium hydroxide (NaOH, ≥ 98%, Cat. No. 345678) were obtained from Merck. All solutions were prepared in double‐distilled water.

### 2.2. Synthesis of Mg‐Doped Ag_2_O NPs

**Figure 1:**
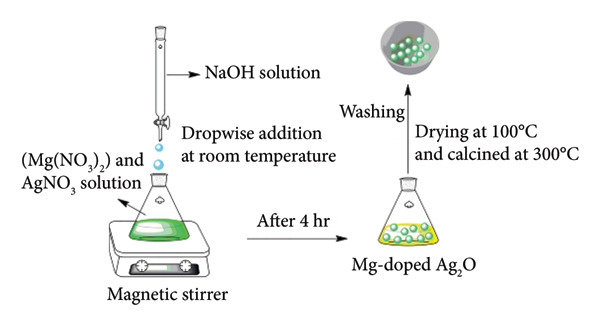
The schematic procedure for the synthesis of Mg‐doped Ag_2_O nanoparticles.

Mg‐doped Ag_2_O nanoparticles were synthesized via the coprecipitation method (illustrated in Figure 1). Briefly, a 0.07 M solution of magnesium nitrate (0.44 g in 50 mL water) was mixed with a 0.7 M solution of silver nitrate (5.94 g in 50 mL water) under continuous stirring. Separately, a 1.4 M solution of sodium hydroxide (2.8 g in 50 mL water) was prepared and added dropwise to the metal nitrate mixture with continuous stirring for 4 h at room temperature. The resulting suspension was allowed to stand undisturbed for 10 h to ensure complete precipitation. The obtained precipitates were repeatedly washed with double‐distilled water to remove residual ions, followed by drying at 100°C. The dried powder was subsequently calcined at 300°C for 3 h in air using a muffle furnace (MTI KSL‐1100X, Nabertherm LE series) at a heating rate of 5°C min^−1^.

### 2.3. Characterization of Mg‐Doped Ag_2_O NPs

The morphological features of the synthesized nanoparticles were examined using scanning electron microscopy (SEM; JEOL JSM‐5800) with a resolution of 3.0 nm operated at 30 kV. The crystalline structure was analyzed by X‐ray diffraction (XRD) using a JEOL JDX‐5332 diffractometer equipped with Cu Kα radiation (*λ* = 1.5406 Å), scanned over a 2*θ* range of 10°–80° at a rate of 2° min^−1^. Fourier‐transform infrared (FTIR) spectra were recorded on a PerkinElmer 95120 spectrophotometer within the range of 4000–400 cm^−1^ at a spectral resolution of 4 cm^−1^. Optical properties were evaluated using a Shimadzu UV‐1800 double‐beam spectrophotometer, recording absorption spectra in the wavelength range of 200–800 nm.

### 2.4. Photocatalytic Activity of Synthesized Mg‐Doped Ag_2_O NPs

**Figure 2:**
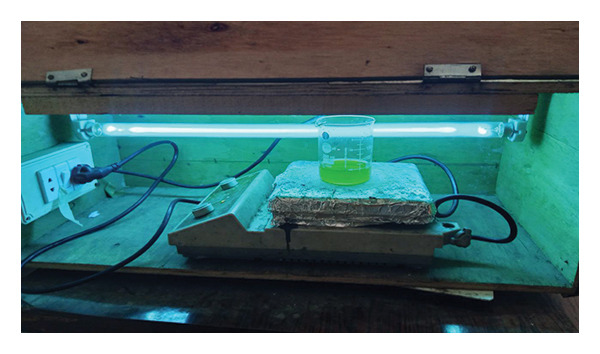
A schematic illustration of the photoreactor used for the decolorization of methyl orange using Mg‐doped Ag_2_O nanoparticles.

Photocatalytic degradation experiments were conducted in a custom‐built reactor (schematic shown in Figure 2). The reactor comprised a quartz beaker placed within a reflective chamber with a 365‐nm UV lamp (intensity 10 mW/cm^2^) positioned 10 cm above the sample surface. The setup was fully enclosed to prevent UV exposure, and all experiments were performed under appropriate shielding and personal protective equipment (PPE). The reaction temperature was monitored and maintained at 25°C ± 1°C without external heating. In a typical run, 0.01 g of Mg‐doped Ag_2_O catalyst was dispersed in 50 mL of 30 ppm MO solution prepared in double‐distilled water and magnetically stirred. Prior to irradiation, the suspension was kept in the dark for 25 min to establish adsorption–desorption equilibrium. Photocatalytic degradation was initiated by irradiating the suspension with the UV lamp for predetermined time intervals. At each interval, 5 mL of the aliquots was withdrawn and centrifuged at 6000 rpm for 5 min, and the supernatant was analyzed by UV–visible spectrophotometry. When required, samples were additionally filtered through a 0.22‐μm membrane filter to prevent catalyst loss.

### 2.5. Degradation Efficiency Calculation

The percentage degradation of MO was calculated using the following:

(1) Degradation=C0CtC0×100,

where *C*_0_ is the initial concentration and *C*_*t*_ is the concentration at irradiation time *t*. Concentrations were determined from UV–Vis absorbance at *λ*_max_ = 464 nm using a preestablished calibration curve.

### 2.6. Kinetic Analysis

The kinetics of photocatalytic degradation of MO were evaluated using pseudo‐first‐order and pseudo‐second‐order models. The pseudo‐first‐order model is expressed as

(2) −dcdt=kAppC,

where *C*_0_ and *C*_*t*_ are the initial and time‐dependent concentrations of the dye, respectively, and *k*_App_ is the apparent rate constant.

The pseudo‐second‐order model is expressed as

(3) 1C−1C0=kApp.t,

where *k*_2_ is the second‐order rate constant. The temperature dependence of the reaction rate was analyzed using the following Arrhenius equation:

(4) k=Ae−EaRT,

where *k* is the rate constant, *A* is the preexponential factor, *E*_*a*_ is the activation energy, *R* is the gas constant, and *T* is the absolute temperature. Activation energy was obtained from the slope of the $\ln k_{{\;v}_{s}}1/T$ plot.

## 3. Results and Discussion

### 3.1. Optical Band Gap of Ag_2_O and Mg‐Doped Ag_2_O NPs

The UV–Vis absorption spectrum of Mg‐doped Ag_2_O nanoparticles is presented in Figure 3(a). The undoped Ag_2_O exhibited an absorption edge around ∼425 nm, corresponding to a band gap of 2.92 eV, while Mg‐doped Ag_2_O showed a redshift to ∼418 nm, resulting in a narrow band gap of 2.60 eV. The absorption peak at 418 nm shows the effect of Mg interaction on the electronic structure of Ag_2_O, particularly the transfer of electrons from the valence band to the conduction band. This transition corresponds to the light absorption at this wavelength, indicating the presence of energy levels, which allow such transitions. The band gap energy (Eg) of the Mg‐doped Ag_2_O nanoparticles was precisely determined using the Tauc plot method (Figure 3(b)). By plotting (*αhν*)^2^ against *h* *ν* and examining the intercept on the energy axis, the band gap value was determined to be 2.60 eV. The minimum energy required for the transition of electrons from the valence band to the conduction band which is considered as a key factor for understanding the optical and electronic behavior of the material. The results show that the band gap energy decreased upon Mg doping due to Mg^2+^ ions facilitating electron excitation. The electronic alternation increases visible‐light absorption and is expected to suppress electron–hole recombination, thereby improving photocatalytic performance [25].

Figure 3(a) UV–Vis absorption spectrum and (b) Tauc plot of Mg‐doped Ag_2_O photocatalysts.

**Figure:**
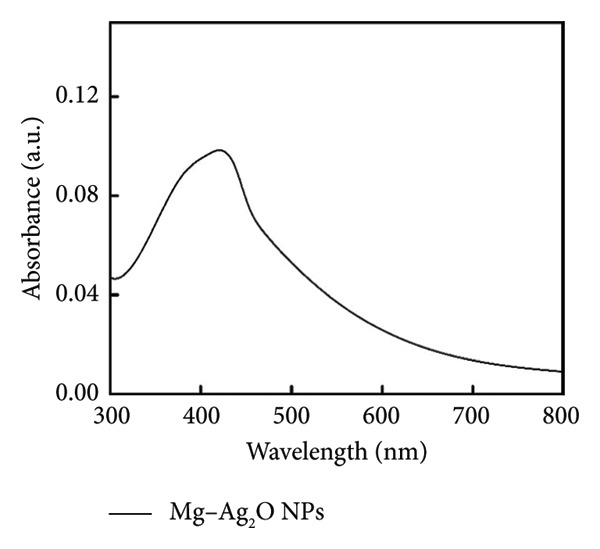
(a)

**Figure:**
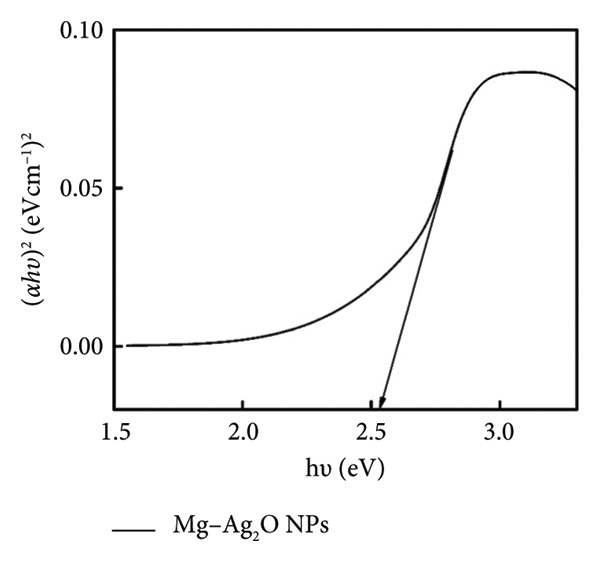
(b)

### 3.2. FTIR Spectroscopic Analysis

**Figure 4:**
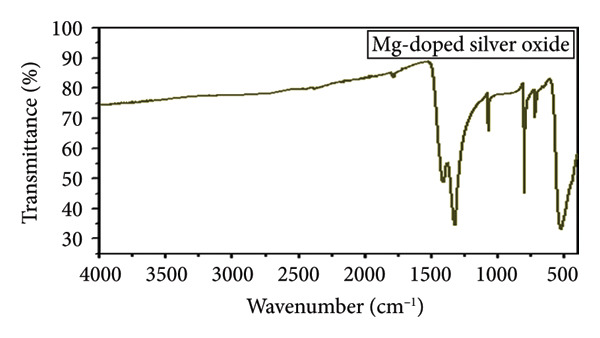
FTIR spectrum of Mg‐doped Ag_2_O NPs.

The FTIR spectra of Mg‐doped Ag_2_O nanoparticle are shown in Figure 4. Mg‐doped Ag2O sample exhibited characteristic absorption bands associated with Ag–O stretching vibrations (∼721 cm^−1^) and adsorbed water (H–O–H bending at ∼1409 cm^−1^). The peak at ∼1323 cm^−1^ corresponds to NO_3_^−^ ions, likely originating from precursor residues. Importantly, a new absorption band attributed to the Mg–O stretching mode appeared at ∼523 cm^−1^ for Mg‐doped Ag_2_O, accompanied by a slight shift (∼532 cm^−1^) relative to Mg‐doped Ag_2_O. This shift confirms the successful incorporation of Mg^2+^ into the Ag_2_O lattice. The reduced intensity suggests partial substitution without major lattice distortion.

### 3.3. SEM and EDX Analysis

**Figure 6:**
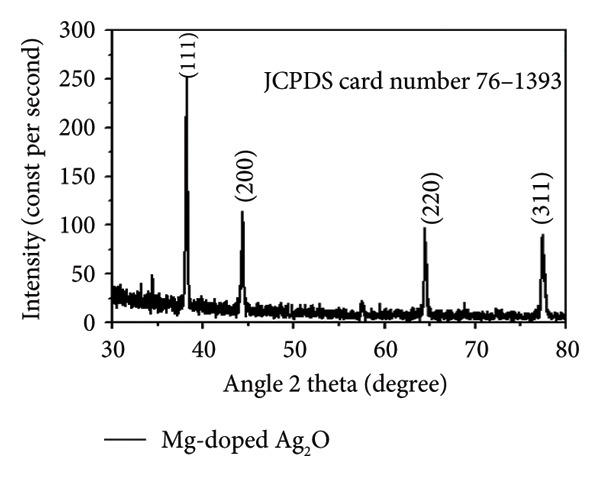
XRD analysis using Mg‐doped Ag_2_O nanoparticles.

SEM micrographs (Figure 5(a)) reveal that undoped Ag_2_O nanoparticles tend to form irregularly shaped agglomerates, whereas Mg‐doped Ag_2_O exhibits a more uniform spherical and well‐defined spherical morphology. Quantitative image analysis indicated an average particle size of 24.1 ± 3.2 nm for Mg‐doped Ag_2_O, slightly smaller than that of undoped Ag_2_O (27.8 ± 3.6 nm), suggesting that Mg doping suppresses particle growth during synthesis. The particle size distribution histogram derived from SEM images is shown in Figure 5(b). Higher‐resolution SEM images (Figure 6(a)) further confirm the spherical morphology, while the corresponding histogram (Figure 6(b)) shows a mean particle size of 23.9 ± 2.8 nm, which is in good agreement with the crystallite size estimated from XRD analysis.

Figure 5(a) EDX image and (b) SEM image of Mg‐doped Ag_2_O NPs.

**Figure:**
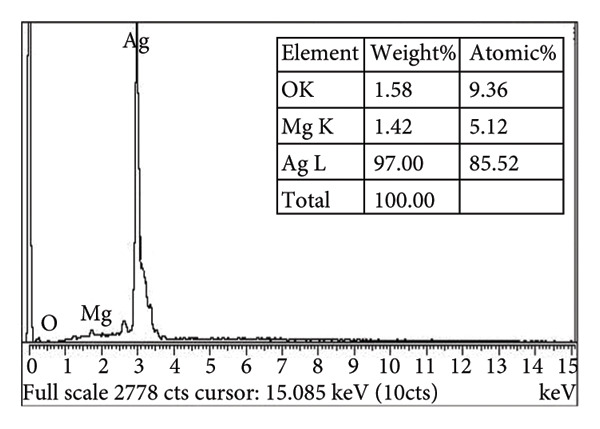
(a)

**Figure:**
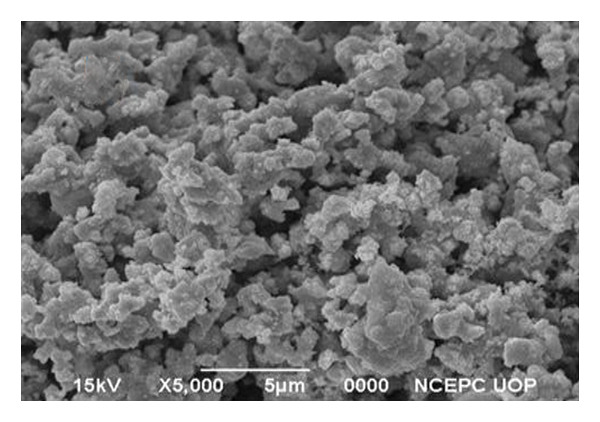
(b)

### 3.4. XRD Analysis of Mg‐Doped Ag_2_O Nanoparticles

The analysis of Mg‐doped Ag_2_O nanoparticles, synthesized through the coprecipitation technique, is elucidated through the XRD pattern presented in Figure 6. A notable feature in the XRD pattern is the presence of a highly intense diffraction peak at 38°, indicating the orientation of Mg‐doped Ag_2_O nanoparticles along the (111) axes. This distinctive diffraction pattern is indicative of a cubic phase structure for the Mg‐doped Ag_2_O nanoparticles. The cubic phase structure is further confirmed by the agreement of the observed peaks at 2Ɵ = 38°, 44°, 64°, and 77° with the expected diffraction peaks for (111), (200), (220), and (311) crystallographic planes, respectively. This alignment with specific crystallographic planes supports the identification of the cubic phase structure in Mg‐doped Ag_2_O nanoparticles. Moreover, the XRD data closely resemble the reference pattern provided by the JCPDS card # 76‐1393. This correlation reinforces the structural integrity and composition of the synthesized Mg‐doped Ag_2_O nanoparticles, establishing their crystalline nature and confirming the successful incorporation of magnesium into the silver oxide matrix. The presented XRD analysis offers valuable insights into the structural characteristics and phase identification of the Mg‐doped Ag_2_O nanoparticles [26]:

(5) D=KʎβcosƟ,

(6) D=0.9ʎβcosƟ,

(7) D=0.91.54×0.350.0174××cos18.12,

(8) D=23.87 nm,

where *K* is the crystallite shape factor constant (0.9), β is the full width at half maximum (FWHM) in radians, θ is Bragg’s diffraction angle, and λ is the X‐ray wavelength for Cu Kα radiation (1.54 Å). Expansive and distinct peaks were seen, indicating the purity and size of the crystallites in the Mg‐doped Ag_2_O NPs [27].

### 3.5. Transmission Electron Microscopic (TEM) Analysis of Mg‐Doped Ag_2_O NPs

**Figure 7:**
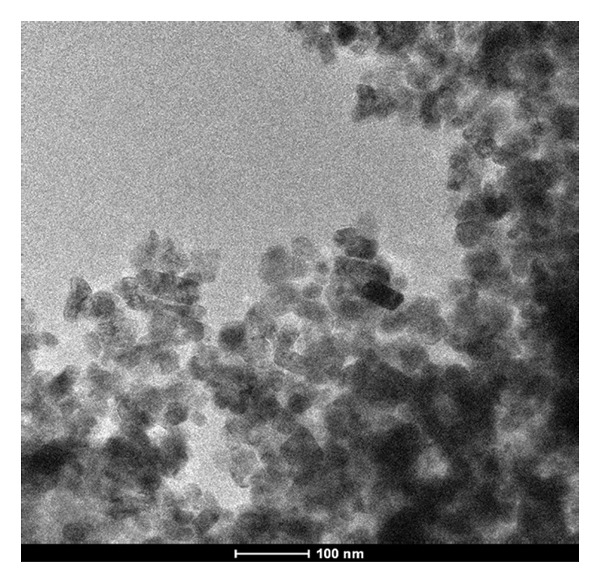
TEM analysis of Mg‐doped Ag_2_O NPs.

The morphology of Mg‐doped Ag_2_O nanoparticles was examined using TEM, as shown in Figure 7. The micrograph, captured at a scale of 100 nm, reveals that the nanoparticles possess an approximately spherical morphology with well‐defined boundaries, indicating uniform particle formation and a controlled synthesis process. The TEM image exhibited the distinct structure of the nanoparticles, highlighting their uniformity and clearly defined boundaries. As an essential parameter, it was quantitatively shown that the average particle size was 23.87 nm. This measurement provides insights into the nanoscale dimensions of the Mg‐doped Ag_2_O particles, reinforcing the precision achieved in the synthesis. This consistency confirms that the synthesized Mg‐doped Ag_2_O nanoparticles are highly uniform in size and exhibit excellent nanoscale structural integrity.

### 3.6. Photoluminescence (PL) Study

**Figure 8:**
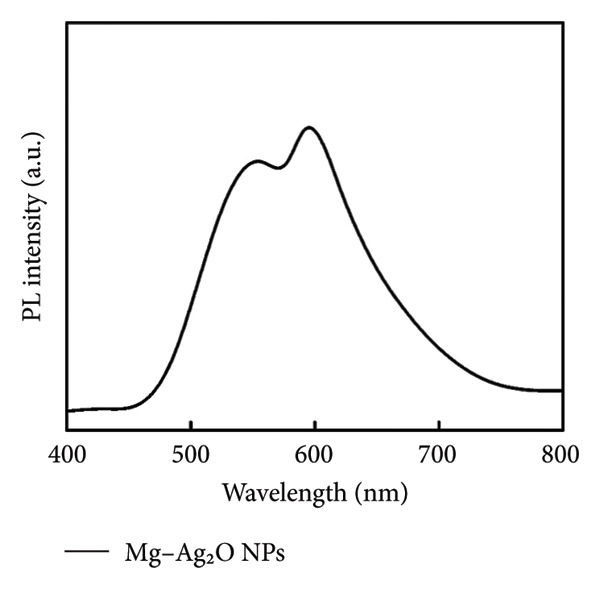
Photoluminescence study of Mg‐doped Ag_2_O NPs.

PL spectroscopy serves as an effective technique for evaluating the charge carrier separation efficiency in semiconductor materials. Figure 8 illustrates the PL spectra of the produced Ag_2_O nanoparticles, recorded under room temperature conditions. The undoped Ag_2_O nanoparticles exhibit two distinct emission peaks at 552 and 589 nm, which are attributed to defect‐related transitions associated with oxygen vacancies and surface states. This diminished PL intensity signifies a lower recombination rate of photogenerated electron–hole pairs, suggesting that Mg doping effectively enhances the charge separation efficiency. Such behavior indicates that Mg incorporation into the Ag_2_O lattice improves its potential photocatalytic performance by minimizing charge carrier recombination losses.

### 3.7. Point of Zero Charge (PZC)

**Figure 9:**
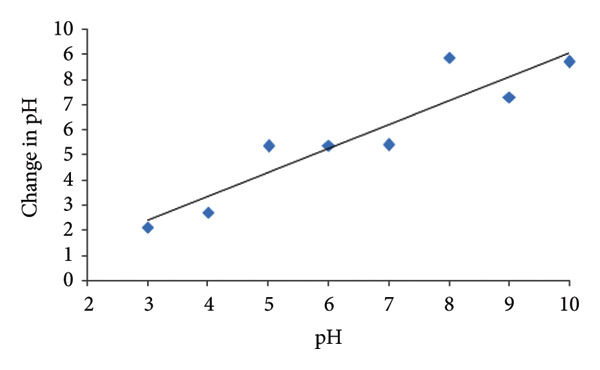
Point of zero charge of Mg‐doped Ag_2_O NPs.

The PZC of Mg‐doped Ag_2_O nanoparticles was determined to evaluate the surface charge behavior under varying pH conditions. Eight separate 0.1 M KNO_3_ solutions were prepared, and their initial pH (pH_0_) values were adjusted between 3 and 10 using 0.01 M HCl or 0.01 M NaOH. After adding 18 mg of Mg‐doped Ag_2_O to the solutions, they were stirred for 24 h at 60 rpm using a magnetic stirrer. After equilibration, the solutions were filtered, and their final pH values were recorded. A plot of pH_0_ versus the change in pH (ΔpH = pH_final − pH_0_) was constructed to determine the PZC, as shown in Figure 9. The intersection point where ΔpH equals zero corresponds to the PZC value. For the Mg‐doped Ag_2_O nanoparticles, the PZC was found to be 6.8, indicating that the surface becomes positively charged at pH < 6.8 and negatively charged at pH > 6.8. At pH < PZC, the catalyst surface is positively charged, enhancing the adsorption of anionic MO dye through electrostatic attraction; at pH > PZC, electrostatic repulsion reduces adsorption efficiency. These findings correlate directly with the pH‐dependent photocatalytic results in Section 3.9.

### 3.8. Effect of Concentration and Time

The influence of initial dye concentration and irradiation time on the photocatalytic degradation of MO using Mg‐doped Ag_2_O was systematically investigated. Dye concentrations of 30, 35, 40, 45, and 50 ppm were exposed to UV radiation in the presence of 0.01 g of catalyst Mg‐doped Ag_2_O. The variations in the percentage of MO dye degradation during various time intervals are displayed in Figures 10(a) and 10(b). The results clearly show that the degradation efficiency increased continuously with illumination time. After 150 min, the photodegradation efficiency of Mg‐doped Ag_2_O was found to be 96.10%. Table 1 shows percent degradation of MO with different catalysts in the literature and the present study.

Figure 10(a) Effect of initial dye concentration on methyl orange dye degradation and (b) effect of time on methyl orange dye degradation.

**Figure:**
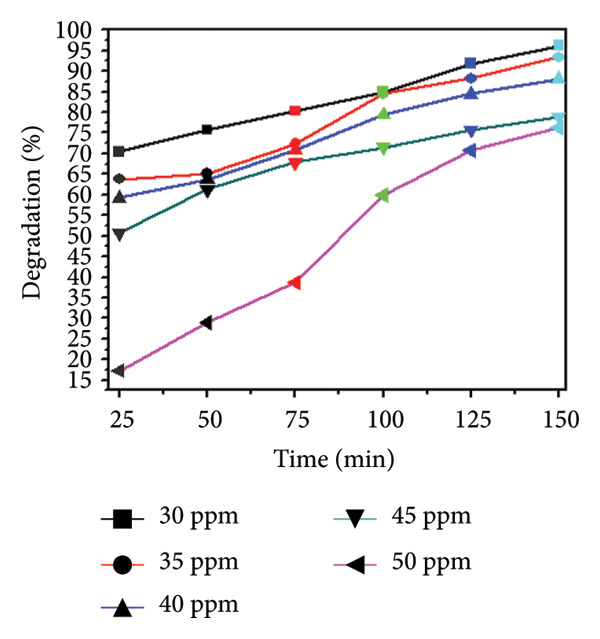
(a)

**Figure:**
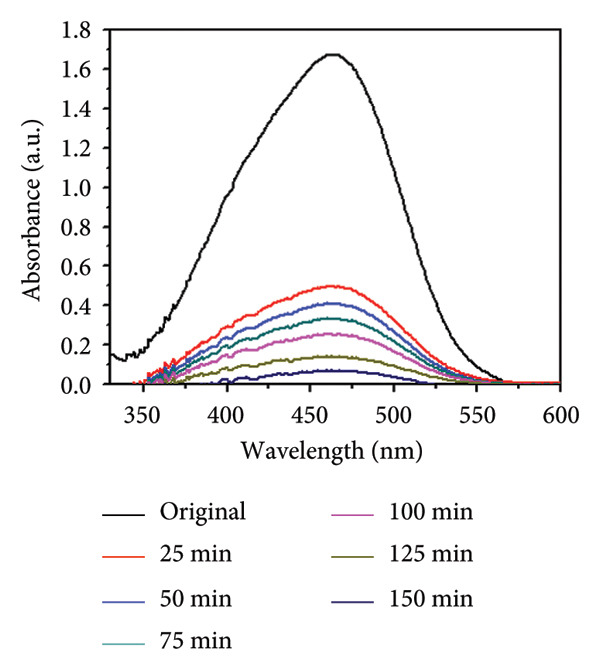
(b)

**Table 1:** Percent degradation of methyl orange with different catalysts in the literature and the present study.

Dye	Nanoparticles	Time	Degradation
Methyl orange	α‐Fe_2_O_3_	100 min	95.31% [28]
	Al‐doped ZnO	40 min	99% [29]
	Sn‐doped TiO_2_	3 h	90% [30]
	CoFe_2_O_4_–SiO_2_–TiO_2_	160 min	93.46% [31]
	Mg‐doped Ag_2_O	150 min	93% present study

This behavior can be attributed to the increased availability of active surface sites on the catalyst at lower dye concentrations, facilitating effective adsorption and photon absorption. As dye concentration increases, the quantity of dye molecules adsorbed on the catalyst surface also increases. This high concentration diminishes the efficacy of the photocatalyst by reducing the number of molecules needed to absorb light photons and subsequently reach the catalyst surface [8, 32]. As a result, MO dye degraded more effectively at 30 ppm.

### 3.9. Catalyst Dose

The effect of catalyst dosage on the photocatalytic degradation efficiency of MO using Mg‐doped Ag_2_O nanoparticles was investigated under UV–visible irradiation (wavelength range: 200–800 nm) for 150 min. The results are illustrated in Figures 11(a) and 11(b). When the reaction was conducted without the catalyst, the self‐degradation of MO was almost negligible under visible light illumination. The rate of decomposition has been markedly accelerated in the presence of Mg‐doped Ag_2_O nanoparticles. After 150 min, the degradation efficiency increased with increasing catalyst doses of Mg‐doped Ag_2_O nanoparticles, peaking at 93.22% at 0.4 g. Thereafter, the degradation efficiency decreased with further catalyst additions. Heterogeneous photocatalysis is characterized by an increase in dye degradation with increasing catalyst concentration. The most common explanation offered for this is that a higher amount of catalyst results in more active sites on the photocatalyst surface, which in turn produces more hydroxyl radicals that can cause dye solution discoloration. However, the degradation rate falls when the catalyst concentration rises over the optimal level because too much catalyst prevents light penetration. The photocatalytic system’s major oxidant, hydroxyl radical, diminished, and as a result, the effectiveness of the degradation dropped [24, 31, 33].

Figure 11(a) Effect of catalyst’s dose on methyl orange dye degradation and (b) the absorbance spectrum of methyl orange dye using Mg‐doped Ag_2_O NPs.

**Figure:**
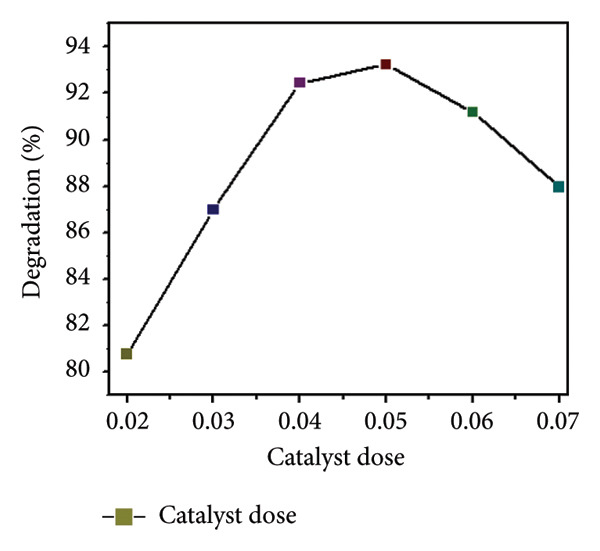
(a)

**Figure:**
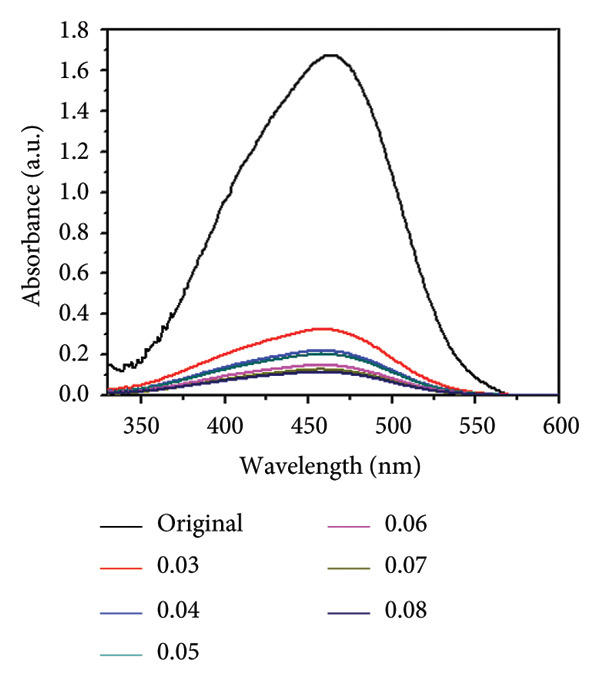
(b)

### 3.10. pH Study Using Mg‐Doped Ag_2_O NPs

The influence of solution pH on the photocatalytic degradation of MO using Mg‐doped Ag_2_O nanoparticles was systematically examined, as shown in Figures 12(a) and 12(b). The results revealed that pH had a considerable effect on the degradation efficiency. At a pH of 7, the greatest rate of MO elimination was achieved [33]. Nevertheless, it has been reported in the literature that using Mg‐doped Ag_2_O nanoparticles to catalyze the photodegradation of MO led to a greater rate of elimination at lower pH values. Various studies on the impact of pH on the photodegradation of MO using Ag_2_O nanoparticles doped with Mg have been conducted. Although neutral conditions (pH 7) yielded the highest degradation efficiency, slightly acidic environments (pH < 6.8) also favored adsorption due to enhanced surface–dye interactions. At highly basic conditions, the repulsive forces between the catalyst surface and dye molecules hindered adsorption and reduced photocatalytic performance. These findings are consistent with previously reported studies, confirming that surface charge interactions governed by pH play a pivotal role in the photocatalytic degradation process.

Figure 12(a) Effect of different pH values on methyl orange dye degradation and (b) the absorbance spectrum of methyl orange dye using Mg‐doped Ag_2_O NPs.

**Figure:**
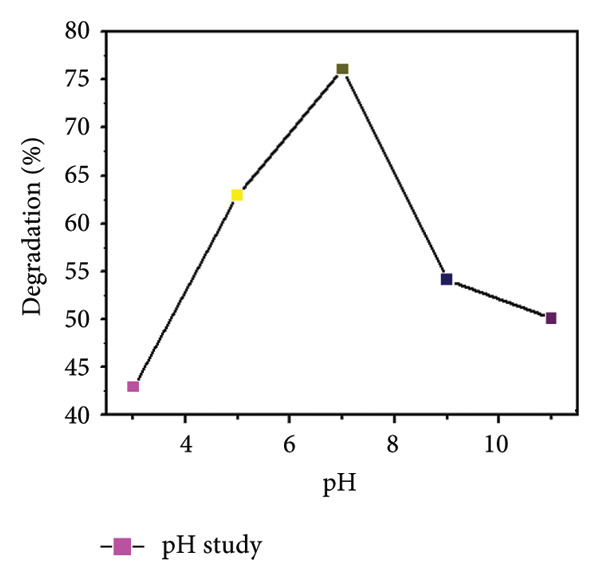
(a)

**Figure:**
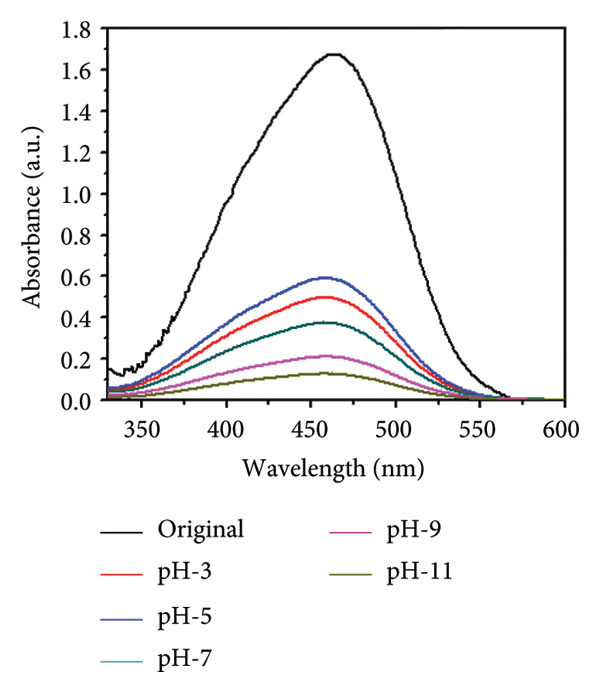
(b)

An important aspect of sustainable heterogeneous catalysis is the catalyst’s reusability. To determine the recyclability of the synthesized Mg‐doped Ag_2_O nanoparticles, the used catalyst was collected at each run, washed with an ethanol–water solution, dried, and reused for the photodegradation of MO under different reaction conditions. Figures 13(a) and 13(b) indicate that the synthesized catalyst has high photocatalytic activity with minor performance loss. Particularly, Mg‐doped Ag_2_O maintained 94%, 92%, 90%, and 88% of its initial activity after four cycles. The minimal loss indicates suitable structural stability and reusability, providing its potential for wastewater treatment application [29].

Figure 13Photocatalytic degradation of MO dye: (a) percent degradation and (b) absorbance spectra of MO dye with reusable catalyst Mg‐doped Ag_2_O.

**Figure:**
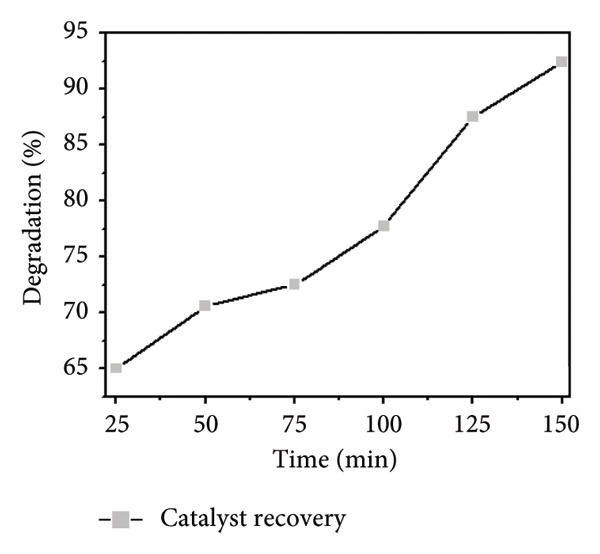
(a)

**Figure:**
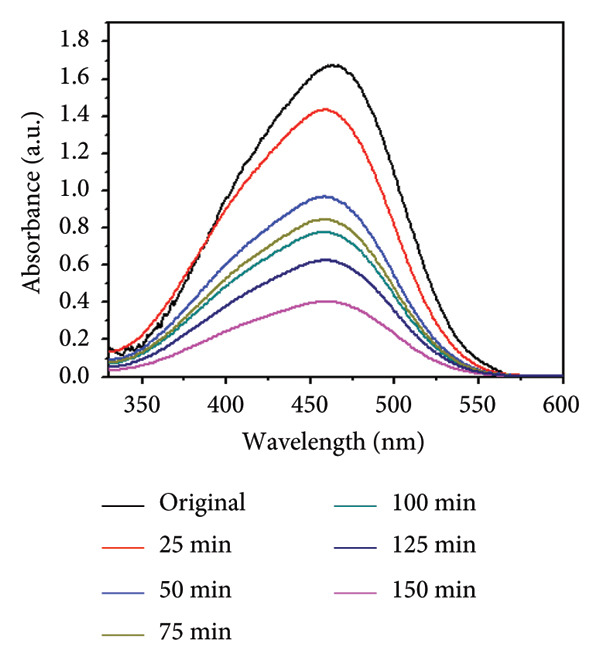
(b)

### 3.11. Effect of Temperature

Temperature is an important parameter that could affect photocatalytic reaction time. The impact of temperature (65–75°C) was assessed by using 0.01 g catalyst and 30 ppm of MO dye. The results are shown in Figures 14(a), 14(b), 14(c), and 14(d), and the degradation of MO dye increased from 85.32% to 88.91% with increasing temperature. The highest degradation at high temperatures was due to improved particle activation, which maximizes the catalyst’s contact with dye molecules and accelerates the dye’s conversion to carbon dioxide and water molecules [29]. In the previous study by Rauf and Ashraf [30], methyl orange dye degradation efficiency increased from 64% at 20°C to 98% at 80°C.

Figure 14(a) Effect of different temperatures on methyl orange dye degradation and the absorbance spectrum of methyl orange dye at (b) 65°C, (c) 70°C, and (d) 75°C using Mg‐doped Ag_2_O nanoparticles.

**Figure:**
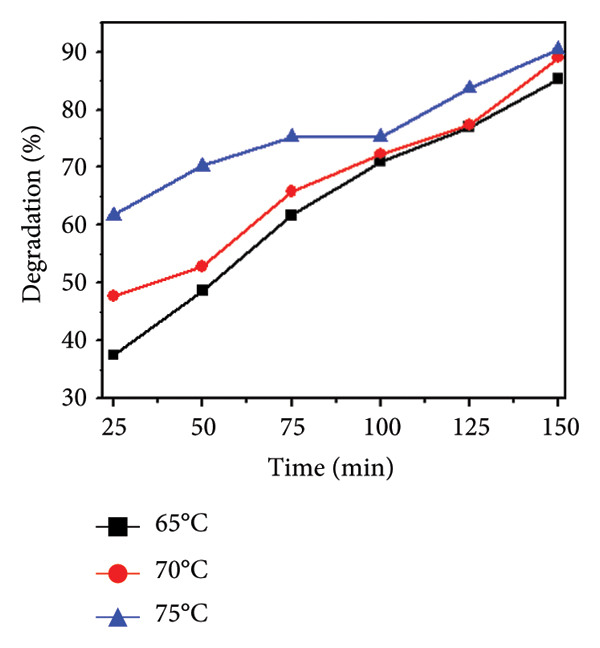
(a)

**Figure:**
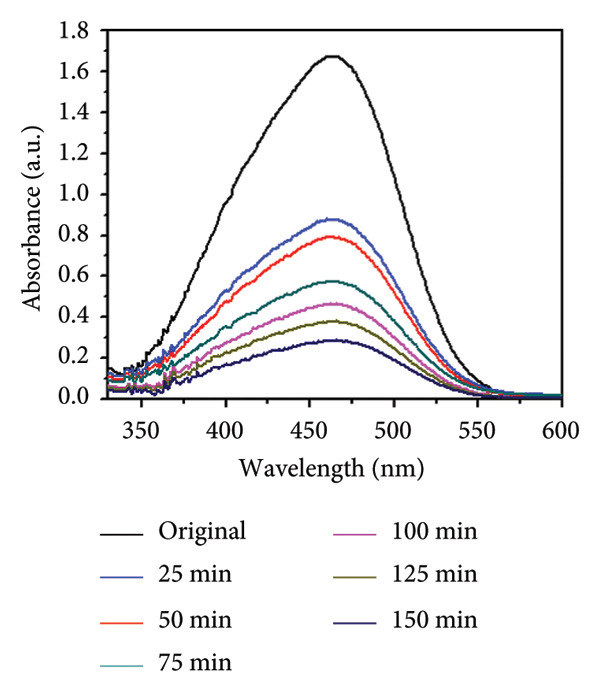
(b)

**Figure:**
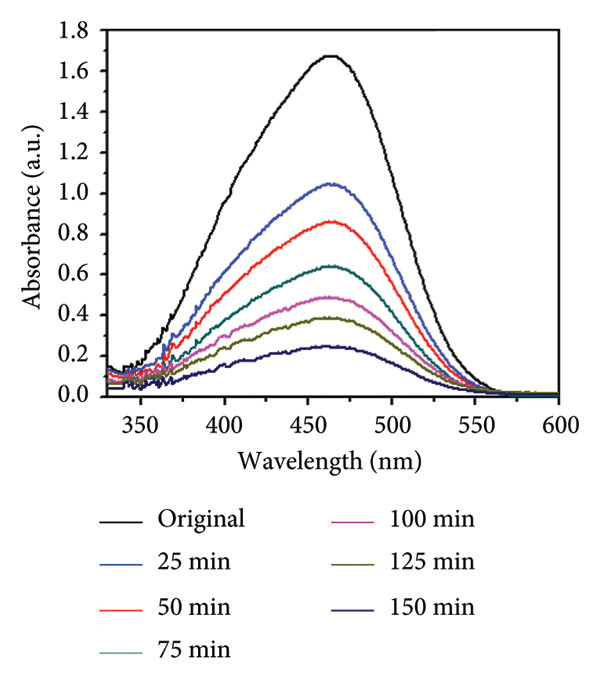
(c)

**Figure:**
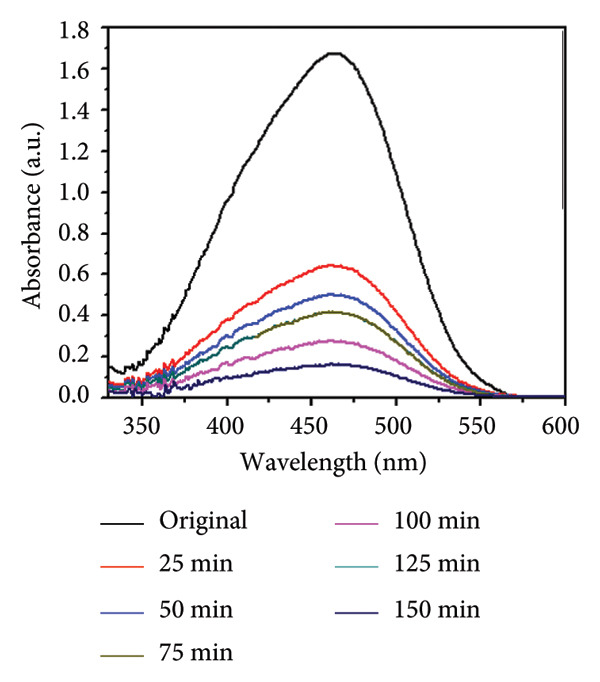
(d)

### 3.12. Kinetic Analysis

To evaluate the reaction kinetics, the experimental data were fitted to pseudo‐first‐ and pseudo‐second‐order models at different initial dye concentrations (30–50 mg L^−1^). The corresponding linear plots are shown in Figures 15(a) and 15(b). The apparent rate constants (*K*_App_) and correlation coefficients (*R* ^2^) are listed in Table 2. The data show that the degradation process follows predominantly the pseudo‐first‐order model, indicating that the surface reaction between active sites and dye molecules governs the overall rate [30].

Figure 15Kinetic analysis of methyl orange degradation using Mg‐doped Ag_2_O nanoparticles: (a, b) pseudo‐first‐ and pseudo‐second‐order kinetic models at varying dye concentrations and (c, d) pseudo‐first‐ and pseudo‐second‐order kinetic models at different temperatures.

**Figure:**
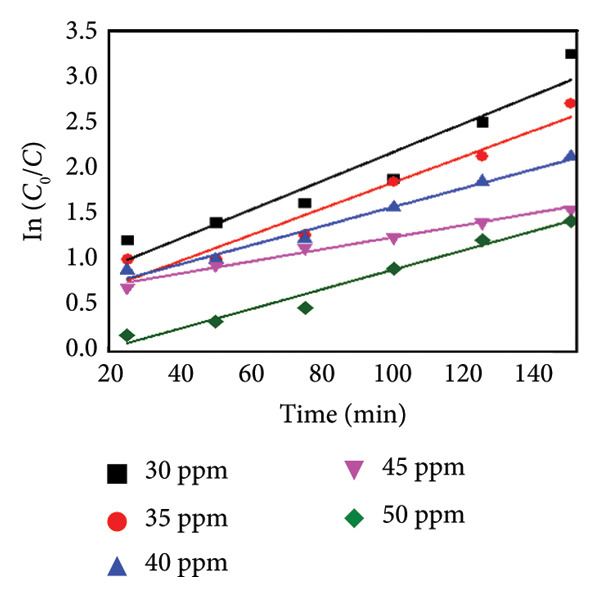
(a)

**Figure:**
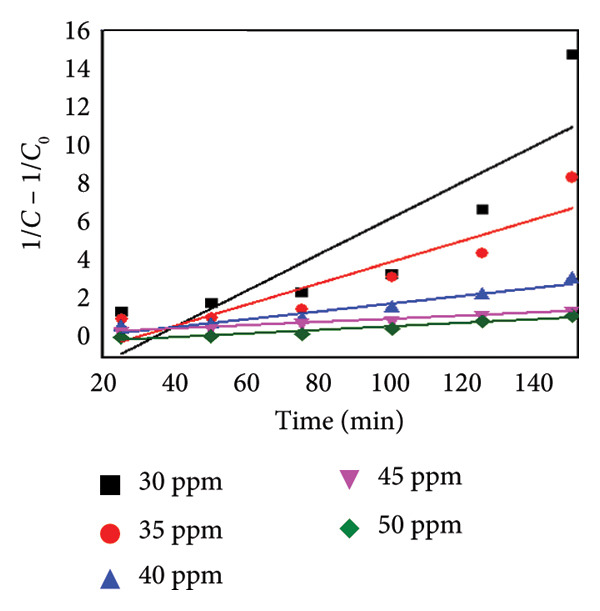
(b)

**Figure:**
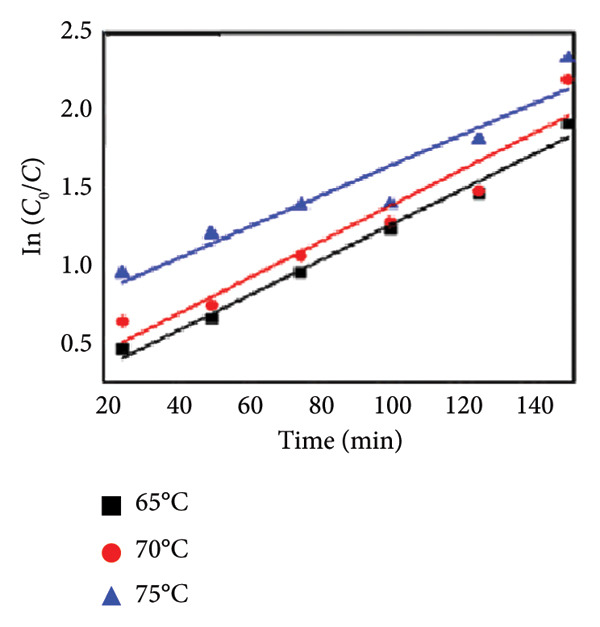
(c)

**Figure:**
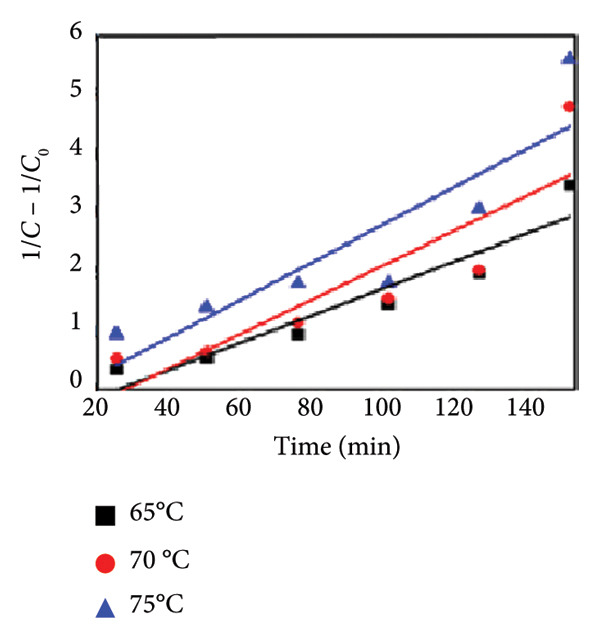
(d)

**Table 2:** Values of the kinetic constant parameter for photocatalytic degradation at various dye concentrations.

Initial concentration (ppm)		First‐order kinetics		Second‐order kinetics	
		**K**_**App**_	**R** ^2^	**K**_**App**_	**R** ^2^
Mg‐doped Ag_2_O	30 ppm	0.01564	0.22737	0.09417	2.6949
	35 ppm	0.01421	0.17592	0.05546	1.20676
	40 ppm	0.01065	0.06778	0.02037	0.26078
	45 ppm	0.01036	0.03726	0.0093	0.02475
	50 ppm	0.00659	0.08496	0.00837	0.00121

**Temperature** °**C**	**First-order kinetics**		**Second-order kinetics**		**Activation energy (kJ/mol)**
	**K**_**App**_	**R** ^2^	**K**_**App**_	**R** ^2^	**13.96**

Mg‐doped Ag_2_O	65	0.02335	0.56879	0.00995	0.0643
	70	0.02953	0.78569	0.01135	0.1628
	75	0.03226	0.36999	0.01162	0.1565

**Figure 16:**
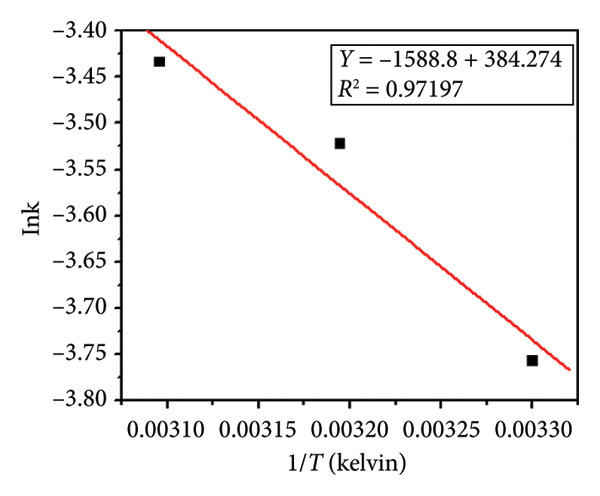
Outcomes of applying the Arrhenius equation to the experimental data with Ag_2_O nanoparticles doped with magnesium.

The effect of temperature also on the apparent constant rate was examined, as shown in Figures 15(c), and 15(d). The result showed the reaction rate improved as the temperature increases from 65°C to 75°C. The activation energy (*E*_*a*_) was achieved from the Arrhenius plot (Figure 16) and was found to be 13.19 kJ mol^−1^. The strong linear correlation (*R* ^2^ = 0.972) shows that the photocatalytic process follows Arrhenius behavior, confirming its temperature‐dependent kinetics. Table 2 summarizes the values of the kinetic constant parameter for photocatalytic degradation at different temperatures.

### 3.13. Dye Mechanism

The photocatalytic degradation of MO over Mg–Ag_2_O follows the conventional semiconductor mechanism, but Mg doping enhances performance by modulating band gap, charge separation, and surface interactions. Upon illumination with photons of energy ≥ 2.60 eV, electrons are promoted from the valence band to the conduction band, leaving holes behind.

(9) Mg doped Ag2O+hvlight⟶e−+h+

**Figure 17:**
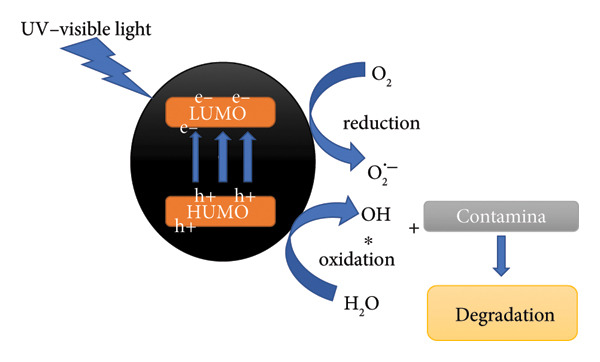
Mechanism for the photocatalytic degradation of methyl orange over Mg‐doped Ag_2_O nanoparticles.

The excited electrons reduce dissolved oxygen to superoxide radicals (•O_2_^-^), while holes oxidize water or hydroxide to hydroxyl radicals (•OH), as illustrated in Figure 17 [33].

(10) e−+O2⟶.O2−

(11) OH+dye molecules⟶degraded products

both of which are highly reactive toward dye molecules. MO adsorbed on the catalyst surface undergoes oxidative degradation to intermediates and mineralizes into CO_2_ and H_2_O [28].

(12) O2−+dye molecules⟶degraded products

## 4. Conclusion

By applying coprecipitation techniques, Mg‐doped Ag_2_O NPs were successfully synthesized. The average particle size of the synthesized materials was 15 nm. The synthesized nanoparticles were used for the degradation of MO. The photodegradation study revealed that the an increase in time, temperature, and catalyst dose, the rate of degradation increased, while it decreased with an increase in the initial concentration of the dye. Photodegradation of MO over Mg‐doped Ag_2_O followed first‐order reaction kinetics. The reusability study showed that the recovered catalyst could be used for the degradation of the same dye many times. Mg doping in Ag_2_O effectively narrowed the band gap from 2.92 to 2.60 eV, improved visible‐light absorption, and reduced electron–hole recombination. The catalyst showed excellent photocatalytic activity (96% in 150 min), stability (88% after four cycles), and favorable PZC characteristics for pH tuning. These results highlight Mg‐doped Ag_2_O as a promising photocatalyst for dye wastewater treatment.

## Ethics Statement

This study did not involve the use of human or animal subjects; therefore, ethics clearance was not required.

## Disclosure

All authors have reviewed the final manuscript and agree to be accountable for all aspects of the work presented.

## Conflicts of Interest

The authors declare no conflicts of interest.

## Author Contributions

All authors have contributed to this study at different stages. Gul Asimullah Khan Nabi: study design, method design, analytical protocol design, writing, reviewing, and editing.

Sajjad Hussain and Mati Ullah: experimental assistant and discussion during writing and reviewing.

Gul Asimullah Khan Nabi, Sajjad Hussain, and Mati Ullah: characterization of the materials and data analysis of the results.

Shohreh Azizi, Ilunga Kamika, and Malik Maaza: reviewing and editing.

## Funding

The authors did not receive support from any organization for the submitted work.

## Data Availability

All data supporting the findings of this work are presented within the article; no supporting information was used.
